# Pharmacist Expectations of Telepharmacy Services in Community Pharmacies in Indonesia

**DOI:** 10.1089/tmr.2024.0049

**Published:** 2025-01-10

**Authors:** Imam Fathorrahman, Umi Athijah, Andi Hermansyah, Abdul Rahem

**Affiliations:** ^1^ Doctoral Programme Student, Faculty of Pharmacy, Universitas Airlangga, Surabaya, Indonesia.; ^2^ Faculty of Pharmacy, Universitas Airlangga, Surabaya, Indonesia.

**Keywords:** telehealth, telemedicine, telepharmacy, digital health

## Abstract

**Background:**

The introduction of new digital-based services such as telehealth, telemedicine, telepharmacy, and other digital health services is expected to continue to evolve, both for the benefit of science. Increasing pharmacists’ roles in pharmaceutical services is critical and strategic in accelerating and recovering public health in the digital era because pharmacists can ensure the availability of appropriate, safe, and effective drugs while also monitoring the rational use of drugs to achieve therapeutic goals.

**Objective:**

This study will look at patient and pharmacist preferences for telepharmacy. This study examines pharmacist preferences for telepharmacy.

**Material and Methods:**

This study includes a sample of 457 Indonesian pharmacists. This cross-sectional study was carried out on pharmacists throughout Indonesia in December 2023. The tool utilized was a questionnaire with an online data collection mechanism. The online questionnaire was designed to capture pharmacists' expectations for existing telepharmacy service practices using the inclusion criteria listed below. This study followed the standards of the Declaration of Helsinki, and electronic informed participant consent was acquired. The Medical Ethics Committee of the Faculty of Pharmacy, Universitas Airlangga, approved the study.

**Conclusion:**

According to the study’s findings, the vast majority of pharmacists support the extensive and integrated usage of telepharmacy. This is backed by the belief that pharmaceutical services will be a lucrative possibility in the future. Overall, the data show that patients and pharmacists are willing to adopt and incorporate telepharmacy services.

## Introduction

Globalization and digitalization have triggered uncertain, fluctuating, and complex environmental changes in various sectors including the health sector. The emergence of new digital-based services such as *telehealth*, *telemedicine*, *telepharmacy*, and various other digital health services is predicted to continue to experience sustainable development for both the benefit of science and business. The development of the *internet of things*, *artificial intelligence*, *automation*, *blockchain*, and *other smart digital* also encourages more health care digitalization, comfortable and personalized.

The use of technology has become a daily necessity in our lives, more than just online communication and social media. Despite the fear and anxiety generated by digitalization, the benefits to humanity are apparent. There are three benefits of information technology, namely (1) digitalization allows a personalized approach to solving problems and customization on demand, (2) digitalization provides a platform to build closer relationships between customers and their communities, and (3) digitalization presents new experiences that are superior and integrated.

In the pharmaceutical service sector, digitalization has also encouraged a change in new work culture with a new format that carries the concept of telepharmacy, which is a trend in various countries. The application of telepharmacy during the COVID-19 pandemic has changed new behaviors that are predicted to become new habits after the pandemic. Based on a study conducted by Rabbani et al.^1^ through four electronic databases, PubMed, Scopus, ProQuest, and Cochrane, regarding the identification of the application of telepharmacy services carried out since the pandemic (December 31, 2019 to May 31, 2022), it is reported that the development and application of telepharmacy models in various places can differ between primary, secondary, and easier service hospitals including pharmaceutical services in hospital pharmacies and communities and special service centers. Telepharmacy provides pharmaceutical services to patients with COVID-19, chronic diseases, HIV infection, cancer, cystic fibrosis, and patients taking anticoagulants. Patient expectations during the pandemic changed significantly, expecting an improvement in pharmaceutical services to be stiffened online, which was triggered by fears of transmission if pharmaceutical services were carried out face-to-face.^2^

Increasing the role of pharmacists in pharmaceutical services is very important and strategic in accelerating and recovering public health in the digital era because fundamentally pharmacists can ensure the availability of appropriate, safe, and effective drugs to monitor the rational use of drugs to achieve the desired therapeutic goals.^2^ The results of the study by Supriyanto and Amrin^3^ also show that pharmaceutical services in hospitals to primary care are areas that have experienced a lot of impact from disruption, so pharmacists need to understand changes in behavior and new expectations expected by patients, especially in terms of access, time and effort needed to be able to interact effectively and efficiently in ensuring the development of therapy results, This is in line with the ultimate goal of pharmaceutical care, which is to achieve an optimal quality of life for patients.

In the areas of medicine selection, order review, dispensing, IV admixture verification, patient counseling and monitoring, and clinical service delivery, telepharmacy has proven to be beneficial. In situations when a pharmacist is not physically present or where pharmacy resources may be a short time, such as in geographically remote ambulatory care clinics and health care institutions, telepharmacy may be very helpful in assisting settings that carry out medication-use activities.^4^

The benefits of telepharmacy in addition to increasing value for pharmacists, telepharmacy can also meet patient needs and expectations, improve interprofessional services, and improve health system efficiency. However, despite these benefits, the main obstacles in the implementation of telepharmacy remain, namely the confidentiality of patient data and the privacy of patient health data information. Various studies related to pharmaceutical services using telepharmacy have been widely conducted including research related to patient counseling services (19 studies), drug order review and drug reconciliation (15 studies), optimization of drug therapy (11 studies), monitoring and management of drug reactions adverse (7 studies), assessment of medication adherence (5 studies), and monitoring of drug-related issues (4 studies). Facts on the ground identify that telepharmacy has proven useful in providing pharmaceutical services to patients, including during the pandemic. However, stronger evidence is needed on the reliability, safety, and effectiveness of telepharmacy compared with conventional face-to-face health care delivery models.

This study aims to find out how pharmacists’ expectations for telepharmacy services carried out in community pharmacies in Indonesia can be taken into consideration in developing telepharmacy service models that are by pharmacists’ expectations of carrying out quality pharmaceutical services.

## Methods

This cross-sectional study was conducted on pharmacists across Indonesia in December 2023. This study was performed in line with the principles of the Declaration of Helsinki and electronic informed patient/participant consent was obtained. The study was approved by the Medical Ethics Committee of the Faculty of Pharmacy Universitas Airlangga.

The online questionnaire was created to determine the picture of pharmacists’ expectations for current telepharmacy service practices based on the following inclusion criteria: running a practice of >3 years in community pharmacies, actively providing direct services to patients in pharmacies at least 5 days a week and willing to be pharmacists. The questionnaire was first distributed to a group of pharmacists and subsequently distributed to other pharmacists according to the inclusion criteria. The questionnaire consists of two main parts: multiple-choice questions and open-ended questions.

Questionnaires are tested for validity and reliability. The questionnaire is valid if the correlation coefficient is >0.3 and reliable if Cronbach’s alpha value is >0.6.^5^ Validity and reliability tests showed that the questionnaire was valid (with a correlation value >0.3) and reliable (with a Cronbach alpha value >0.6). Data analysis in the multiple-choice section was performed using descriptive statistics that focused on the percentage of answers. Open-ended questions are evaluated using thematic analysis by sorting, distilling, and reviewing answers into themes that illustrate recommendations for making telepharmaceutical models.

## Results

This study used a descriptive analysis. This approach was used to develop a comprehension of pharmacists’ characteristics and their expectations for telepharmacy services in community pharmacies. The analytic results are displayed as frequency and narrative distribution tables, as well as percentages based on the number of research subjects. The research was also carried out using the *t*-test to discover correlations between variables. Table 1 shows how pharmacists’ expectations for the implementation of telepharmacy services illustrate. The variables seen in this study were: [1] Pharmacists’ need for telepharmacy; [2] Comprehensive and integrated telepharmacy services; [3] Telepharmacy service ımplementation opportunities; [4] The positive impact of telepharmacy practices on pharmaceutical performance; [5] Pharmacist’s willingness to improve competence in telepharmacy.

**Table 1.** Overview of Pharmacists’ Expectations for Telepharmacy Services in Community Pharmacies

Variable	Number of pharmacists (*n* = 457)	Percentage (%)
Pharmacists’ need for telepharmacy		
Strongly disagree	1	0.2
Disagree	16	3.5
Agree	250	54.7
Totally agree	190	41.6
Comprehensive and integrated telepharmacy services		
Strongly disagree	0	0.0
Disagree	10	2.2
Agree	231	50.5
Totally agree	216	47.3
Telepharmacy service ımplementation opportunities		
Strongly disagree	0	0.0
Disagree	8	1.8
Agree	246	53.8
Totally agree	203	44.4
The positive impact of telepharmacy practices on pharmaceutical performance		
Strongly disagree	0	0.0
Disagree	14	3.1
Agree	261	57.1
Totally agree	182	39.8
Pharmacist’s willingness to improve competence in telepharmacy		
Strongly disagree	0	0
Disagree	6	1.3
Agree	253	55.4
Totally agree	198	43.3

## Discussion

### Demographic characteristics of pharmacists

The demographic details of pharmacists who are interested in taking part in the study are discussed in Table 2. Features have been divided into sections: [1] gender, [2] age (years old), [3] period of service, [4] experience doing telepharmacy.

**Table 2.** Demographic Characteristics of Pharmacists

Pharmacist characteristics	Number of pharmacists (*n* = 457)	Percentage (%)
Gender		
Male	116	25.4
Female	341	74.6
Age (years old)		
20–30	140	30.6
31–40	192	42.0
41–50	94	20.6
51–60	23	5.0
61–70	8	1.8
Period of service		
<3 years	84	18.4
>8 years	176	38.5
3–5 years	119	26.0
6–8 years	78	17.1
Experience doing telepharmacy		
Ever	250	54.7
Never	207	45.3

### Gender

An overview of the demographics of pharmacists by gender is given by the data above, showing that there are 74.6% more female pharmacists than male pharmacists (25.4%).

### Age

According to the age distribution of pharmacists who use telepharmacies, around 30.6% of those polled were in their 20s or 30s. Then, around 42% are between the ages of 31 and 40. When the percentages from the two categories are combined, they form a sizable population with high self-motivation to use telepharmacy services. They actively contribute to the full potential of telepharmacy services by adopting new technologies that improve efficiency and service quality. Approximately 20.6% of the people aged 41 to 50 can adapt to current technologies.

### Period of service

The demographics of pharmacists based on length of service illustrate that as many as 18.4% of pharmacists have <3 years of work experience. This group is a new pharmacist who started practice directly by applying the knowledge they gained in the world of education. Although it has a relatively new experience, this group is a rising star that brings major changes to digital culture. While 26% of pharmacists have a tenure of between 3 and 5 years and as many as 17.1% of pharmacists have a tenure of between 6 and 8 years. The combination of these two groups is ideal because, in addition to being able to adapt quickly, they also have practical experience for a longer period and can develop significant conceptual skills and understanding during that period. The remaining 38.5% of pharmacists have a working period of >8 years which is a group that has very broad work experience in developing the pharmacist profession.

It is clear from these data that pharmacists’ job experiences differ in certain ways. The presence of different distributions during this time of employment may have an impact on their knowledge, abilities, and work style in the pharmacy industry. Comprehending these demographic attributes can aid in formulating suitable training initiatives and career advancement strategies for various demographics of pharmacists.

### Experience doing telepharmacy

More than 54.7% of pharmacists stated that they had practiced telepharmacy services. This shows that most pharmacists have a high commitment to using or providing telepharmacy services. Telepharmacy service activities that are often used are still limited to activities that are very simple including remote consultations, online prescriptions, or patient monitoring using technology.

The remaining 45.3% of pharmacists stated that they have never done telepharmacy with various contributing factors including ignorance of understanding related to the use of technology, more focus on face-to-face services, and lack of opportunities for benefits in telepharmacy practice in their workplace.

From this data, it can be concluded that although most pharmacists already have experience in conducting telepharmacy, there are still some who have not been involved in the practice. This shows that there is diversity in technology adoption among pharmacists. A further understanding of the reasons behind experience or inexperience in telepharmacy can help design appropriate training or education strategies to support the application of this technology in the pharmacist profession. Table 1 shows how the demographic characteristics of pharmacists are based on gender, age, length of service as a pharmacist, and experience using telepharmacy services.

## Overview of pharmacists’ expectations for telepharmacy services in community pharmacies

### Pharmacists’ need for telepharmacy

A small number of pharmacists, about 0.2%, stated that they strongly disagreed with the use of telepharmacy. Perhaps they have concerns or inconveniences related to remote health care using technology. Another small percentage, about 3.5% of pharmacists, stated that they disagreed with the use of telepharmacy. Reasons for disapproval can vary, including a lack of trust in technology or a preference for in-person meetings with health professionals.

Most pharmacists, about 54.7%, agreed with the use of telepharmacy. This shows that there is majority support for the application of technology in providing remote health services. Pharmacists who agree may see the benefits of the convenience and accessibility offered by telepharmacy. About 41.6% even stated that they strongly agreed with the use of telepharmacy. This high approval rate shows that there is great trust and interest in adopting technology for health care. Research conducted by Abu-Farha et al.^6^ found that pharmacists are open to using telepharmacy. However, faults with the service must be solved in order to encourage wider deployment and use. This may increase patient access to pharmaceutical care, particularly for individuals living in Jordan's distant regions.

### Pharmacists need comprehensive and integrated telepharmacy services

The data above reflect pharmacist perceptions and needs for telepharmacy services that are comprehensive and integrated with other health services in the future. No pharmacist stated that they strongly disagreed with the need for comprehensive and integrated telepharmacy services. This could indicate that there was no complete rejection of this idea among pharmacists. A small percentage of pharmacists, about 2.2%, disagreed with the need for comprehensive and integrated telepharmacy services.

Most pharmacists, around 50.5%, agreed with the need for comprehensive and integrated telepharmacy services. This suggests that there is a significant level of support for this concept among pharmacists. They may see potential benefits in improving accessibility, efficiency, and coordination of care through telepharmacy integration. A large number of pharmacists, about 47.3%, even stated that they strongly agree with the need for comprehensive and integrated telepharmacy services. This high percentage indicates strong support for the idea that telepharmacy should be an integral part of the health care system for the foreseeable future.

The majority of pharmacists are aware of and support the idea that comprehensive and integrated telepharmacy services can provide added value in health care provision. It is important to take this understanding into the planning and implementation of telepharmacy services to meet the expectations and needs identified by the pharmacists.

### Opportunities for the implementation of telepharmacy services in the future

The data above provide an overview of pharmacists’ views on opportunities for implementing telepharmacy services in the future. None of the pharmacists stated that they strongly disagreed with the view that opportunities for the future implementation of telepharmacy services are promising. This shows that there was no complete rejection of this idea among pharmacists. A small percentage of pharmacists, about 1.8%, do not fully agree with the view that the opportunities for the future implementation of telepharmacy services are promising. Reasons for disagreement can vary, including distrust of technological developments or uncertainty about the positive impact that telepharmaceuticals can bring.

The majority of pharmacists, about 53.8%, agreed that the opportunities for the future implementation of telepharmacy services are promising. This shows a significant level of support for the development and application of telepharmaceutical technology in providing health services. A large number of pharmacists, about 44.4%, even stated that they strongly agree with the view that the opportunities for the implementation of telepharmacy services in the future are very promising. This high percentage indicates a strong belief in the positive potential and impact that telepharmaceutical use can bring. The majority of pharmacists see promising opportunities in the implementation of telepharmacy services in the future. This reflects optimism toward the role of technology in improving the efficiency, accessibility, and quality of health care. This information can assist in designing strategies and policies that support telepharmacy adoption and implementation. Even though telepharmacy has a lot of potential, there are a few obstacles that need to be overcome before it can fully take hold in the health care system.^7^

### Impact of telepharmacy service practices on pharmaceutical service performance

The data above provide an overview of pharmacists’ views on the impact of implementing telepharmacy service practices on pharmaceutical service performance. No pharmacist stated that they strongly disagreed with the view that the implementation of telepharmacy service practices had an impact on better pharmaceutical service performance. This shows that there is no complete rejection of the idea that telepharmacy can improve the performance of pharmaceutical services. A small percentage of pharmacists, around 3.1%, do not fully agree with the view that the implementation of telepharmacy service practices can have a positive impact on pharmaceutical service performance. The reasons for this disagreement can vary, ranging from uncertainty over technology to concerns over possible negative impacts.

The majority of pharmacists, around 57.1%, agreed that the implementation of telepharmacy service practices has an impact on better pharmaceutical service performance. This shows that most pharmacists see the potential for improvement in providing pharmaceutical services through the use of telepharmacy technology. A large number of pharmacists, around 39.8%, even stated that they strongly agree with the view that the implementation of telepharmacy service practices can have a significant positive impact on pharmaceutical service performance. This high percentage reflects a strong belief in the benefits that can be gained from the integration of telepharmacy into pharmaceutical practice. By increasing the level of pharmaceutical services, it is hoped that it will affect patient compliance. Research conducted by Abughosh et al.^8^ shows that intensified care, including intensified patient counseling, education, and training programs, from a pharmacist can improve medication adherence among patients with *Diabetes* *mellitus*, also known as DM (a metabolic illness in which blood sugar levels stay high) and *hypertension* (high blood pressure).

From this data, it can be concluded that the majority of pharmacists see that the implementation of telepharmacy service practices can improve the performance of pharmaceutical services. This information can be useful in designing and implementing telepharmacy initiatives in the pharmaceutical environment, as well as in shaping views and related policies in this area.

### Willingness of pharmacists to improve their competence in the field of telepharmacy services

The data reflect pharmacists’ level of willingness to improve their competence in the field of telepharmacy services. No pharmacist has expressed that they strongly disagree with the willingness to improve competence in the field of telepharmacy services. This shows that there is no complete rejection of the idea of improving telepharmacy-related skills and knowledge among pharmacists. A small percentage of pharmacists, about 1.3%, do not fully agree with the willingness to improve competence in the field of telepharmacy services. The reasons for this disagreement may be varied, such as discomfort with the technology or lack of confidence in its benefits.

The majority of pharmacists, about 55.4%, agreed to improve competence in the field of telepharmacy services. This suggests that most pharmacists are willing to develop telepharmacy-related skills and knowledge, perhaps because they see its added value or benefits in pharmacy practice. A large number of pharmacists, about 43.3%, even stated that they strongly agreed to improve competence in the field of telepharmacy services. This high percentage reflects a high level of willingness to adopt and develop the skills needed to participate in telepharmacy services.

## Conclusion

The majority of pharmacists (96.5%) tend to agree or strongly agree with the use of telepharmacy. Only a small percentage (3.7%) disagreed. This shows the wide support and acceptance of telepharmacy among pharmacists. Nonetheless, it is worth noting and understanding the reasons behind the level of disapproval to be able to increase the application of this technology. Then, most pharmacists (97.8%) agreed or strongly agreed with the need for comprehensive and integrated telepharmacy services. The high approval rate demonstrates confidence in the potential benefits and improvement of health care quality through telepharmacy integration.

The majority of pharmacists (98.2%) see promising opportunities in the implementation of telepharmacy services in the future. The high level of optimism reflects confidence in the positive contribution of telepharmaceutical technology in improving the efficiency and accessibility of health care. Most pharmacists (96.9%) agree or strongly agree that implementing telepharmacy service practices can positively impact pharmaceutical service performance. The high approval rate indicates recognition of the potential for improving pharmaceutical services through the utilization of telepharmacy. The majority of pharmacists (98.7%) are willing to improve competence in the field of telepharmacy services. This high level of willingness is a positive thing in ensuring that pharmacists can adapt themselves and provide effective services by utilizing telepharmacy technology.

Overall, the results of these data show the support and willingness to adopt and integrate telepharmacy services. Further understanding of the reasons behind lower levels of disapproval and strategies to overcome these barriers can focus on maximizing the benefits of telepharmacy within the health care system.
